# Differentiated pattern of complement system activation between MOG-IgG-associated disease and AQP4-IgG-positive neuromyelitis optica spectrum disorder

**DOI:** 10.3389/fimmu.2024.1320094

**Published:** 2024-03-21

**Authors:** Eun Bin Cho, Ju-Hong Min, Patrick Waters, Miyoung Jeon, Eun-Seon Ju, Ho Jin Kim, Su-Hyun Kim, Ha Young Shin, Sa-Yoon Kang, Young-Min Lim, Sun-Young Oh, Hye Lim Lee, Eunhee Sohn, Sang-Soo Lee, Jeeyoung Oh, Sunyoung Kim, So-Young Huh, Joong-Yang Cho, Jin Myoung Seok, Byung-Jo Kim, Byoung Joon Kim

**Affiliations:** ^1^ Department of Neurology, Gyeongsang Institute of Health Science, Gyeongsang National University, College of Medicine, Jinju, Republic of Korea; ^2^ Department of Neurology, Gyeongsang National University Changwon Hospital, Changwon, Republic of Korea; ^3^ Department of Neurology, Samsung Medical Center, Sungkyunkwan University School of Medicine, Seoul, Republic of Korea; ^4^ Department of Neurology, Neuroscience Center, Samsung Medical Center, Seoul, Republic of Korea; ^5^ Department of Health Sciences and Technology, Samsung Advanced Institute for Health Sciences & Technology (SAIHST), Sungkyunkwan University, Seoul, Republic of Korea; ^6^ Oxford Autoimmune Neurology Group, Nuffield Department of Clinical Neurosciences, John Radcliffe Hospital, University of Oxford, Oxford, United Kingdom; ^7^ Samsung Research Institute of Future Medicine, Seoul, Republic of Korea; ^8^ Department of Neurology, Research Institute and Hospital of National Cancer Center, Goyang, Republic of Korea; ^9^ Department of Neurology, Yonsei University College of Medicine, Seoul, Republic of Korea; ^10^ Department of Neurology, Jeju National University Hospital, Jeju National University School of Medicine, Jeju, Republic of Korea; ^11^ Department of Neurology, Asan Medical Center, University of Ulsan College of Medicine, Seoul, Republic of Korea; ^12^ Department of Neurology, Chonbuk National University Hospital, School of Medicine, Chonbuk National University, Jeonju, Republic of Korea; ^13^ Department of Neurology, Korea University Guro Hospital, Korea University College of Medicine, Seoul, Republic of Korea; ^14^ Department of Neurology, Chungnam National University Hospital, School of Medicine, Chungnam National University, Daejeon, Republic of Korea; ^15^ Department of Neurology, Chungbuk National University Hospital, School of Medicine, Chungbuk National University, Cheongju, Republic of Korea; ^16^ Department of Neurology, Konkuk University Hospital, School of Medicine, Konkuk University, Seoul, Republic of Korea; ^17^ Department of Neurology, Ulsan University Hospital, Ulsan University, College of Medicine, Ulsan, Republic of Korea; ^18^ Department of Neurology, Kosin University Hospital, College of Medicine, Kosin University, Busan, Republic of Korea; ^19^ Department of Neurology, Ilsan Paik Hospital, Inje University College of Medicine, Goyang, Republic of Korea; ^20^ Department of Neurology, Soonchunhyang University Cheonan Hospital, Soonchunhyang University College of Medicine, Cheonan, Republic of Korea; ^21^ Department of Neurology, Korea University Anam Hospital, Korea University College of Medicine, Seoul, Republic of Korea

**Keywords:** myelin oligodendrocyte glycoprotein, neuromyelitis optica spectrum disorder, complement, terminal complement complex (sC5b-9), classical complement cascade, alternative complement activity

## Abstract

**Background:**

Myelin oligodendrocyte glycoprotein antibody (MOG) immunoglobulin G (IgG)-associated disease (MOGAD) has clinical and pathophysiological features that are similar to but distinct from those of aquaporin-4 antibody (AQP4-IgG)-positive neuromyelitis optica spectrum disorders (AQP4-NMOSD). MOG-IgG and AQP4-IgG, mostly of the IgG1 subtype, can both activate the complement system. Therefore, we investigated whether the levels of serum complement components, regulators, and activation products differ between MOGAD and AQP4-NMOSD, and if complement analytes can be utilized to differentiate between these diseases.

**Methods:**

The sera of patients with MOGAD (from during an attack and remission; *N*=19 and *N*=9, respectively) and AQP4-NMOSD (*N*=35 and *N*=17), and healthy controls (*N*=38) were analyzed for C1q-binding circulating immune complex (CIC-C1q), C1 inhibitor (C1-INH), factor H (FH), C3, iC3b, and soluble terminal complement complex (sC5b-9).

**Results:**

In attack samples, the levels of C1-INH, FH, and iC3b were higher in the MOGAD group than in the NMOSD group (all, *p*<0.001), while the level of sC5b-9 was increased only in the NMOSD group. In MOGAD, there were no differences in the concentrations of complement analytes based on disease status. However, within AQP4-NMOSD, remission samples indicated a higher C1-INH level than attack samples (p=0.003). Notably, AQP4-NMOSD patients on medications during attack showed lower levels of iC3b (*p*<0.001) and higher levels of C3 (*p*=0.008), C1-INH (*p*=0.004), and sC5b-9 (*p*<0.001) compared to those not on medication. Among patients not on medication at the time of attack sampling, serum MOG-IgG cell-based assay (CBA) score had a positive correlation with iC3b and C1-INH levels (rho=0.764 and *p*=0.010, and rho=0.629 and *p*=0.049, respectively), and AQP4-IgG CBA score had a positive correlation with C1-INH level (rho=0.836, *p*=0.003).

**Conclusions:**

This study indicates a higher prominence of complement pathway activation and subsequent C3 degradation in MOGAD compared to AQP4-NMOSD. On the other hand, the production of terminal complement complexes (TCC) was found to be more substantial in AQP4-NMOSD than in MOGAD. These findings suggest a strong regulation of the complement system, implying its potential involvement in the pathogenesis of MOGAD through mechanisms that extend beyond TCC formation.

## Introduction

Neuromyelitis optica spectrum disorders (NMOSD) are chronic inflammatory diseases of the central nervous system (CNS) that preferentially affects the optic nerve, spinal cord, and certain brain regions. The discovery of pathogenic antibodies that target aquaporin-4 (AQP4-immunoglobulin G [IgG]) facilitated the recognition of AQP4-IgG positive NMOSD (AQP4-NMOSD) as a distinct disease entity (1). Antibodies against myelin oligodendrocyte glycoprotein (MOG-IgG) were found more recently in a group of patients with demyelinating disease whose clinical features partially overlap with NMOSD and a new disease entity associated with MOG-IgG, called MOG antibody-associated disease (MOGAD), was suggested (2). The clinical phenotypes of MOGAD overlap with those of NMOSD but include a wider range of presenting phenotypes including acute disseminated encephalomyelitis (ADEM), optic neuritis, myelitis, or demyelinating brain lesions; however, its clinical course and prognosis differ from those of AQP4-NMOSD (2, 3).

AQP4 is a major water channel protein in the CNS that is highly expressed in the astrocytic foot processes. Complement-dependent AQP4-IgG-mediated cytotoxicity is a major mechanism of astrocyte damage with secondary oligodendrocyte loss, and these lesions are associated with perivascular deposition of activated complements and inflammatory cell infiltration (4). On the other hand, it has not yet been determined how MOG-IgG contributes to MOGAD pathogenesis. MOG is a minor myelin protein predominantly localized at the outermost layer of the myelin sheaths and oligodendrocyte membranes (5). Recent MOGAD pathologic studies found that ADEM-like perivenous demyelination was predominant and that early-stage lesions included MOG-dominant myelin loss with less oligodendrocyte damage than in AQP4-NMOSD (6). Activated complement and IgG deposition were also found in the active white-matter lesions of MOGAD; however, the frequency and intensity of staining was much lower than that in AQP4-NMOSD, especially in its early stage (6, 7). These findings suggest that the clinical significance of immune response including complement system activation differ between the two diseases. The complexity arises from the blood complement component levels’ potential to serve as indicators of CNS pathobiology, particularly in the context of relapsing disorders. Nevertheless, since antibody production lies within the peripheral circulation, investigating the events occurring there holds the promise of offering valuable insights into the pathophysiology.

In this study we aimed to elucidate differences in complement activation between MOGAD and AQP4-NMOSD by comparing serum levels of complement components, regulators, and activation products.

## Materials and methods

We collected the sera and clinical data of patients with AQP4-NMOSD (8) or MOGAD (9) from 12 tertiary hospitals that participated in the Korean nationwide registry for NMOSD between December 2014 and December 2017. We included 52 NMOSD serum specimens (35 attack and 17 remission samples) and 28 MOGAD serum specimens (19 attack and 9 remission samples). Attack samples were defined as those drawn within 30 days of an attack and remission samples were taken more than 90 days after an attack. Patients were categorized as ‘on medication’ if receiving treatment with steroids or other immunosuppressive agents, irrespective of preventive or acute therapy, at the time of sampling; otherwise, they were labeled as ‘not on medication’. Blood sampling was performed prior to plasmapheresis or intravenous immunoglobulin in all cases of attack samples. Serum samples from 38 healthy controls (HC) who did not have history of acute or chronic disease and had not been taking any medication during the previous 3 months were included as a control group. Control serums were obtained from a single hospital. Only one blood sample was obtained from each participant in this study. Whole blood was collected into serum separating tube and centrifuged for 10 minutes at 2000 rpm. All samples were stored at −80°C within 4 hours after blood sampling prior to the analysis. We kept serum samples on ice during pre-analytical sample handling and avoided freeze-thawing.

AQP4-IgG was evaluated using a cell-based indirect immunofluorescence assay (10). MOG-IgG was determined using an in-house live cell-based immunofluorescence assay (CBA) with an antihuman IgG1-Fc secondary antibody (11). Briefly, human embryonic kidney 293 (HEK293) cells (Korean Cell Line Bank, Seoul, South Korea) were transfected with plasmids encoding human full-length α1-MOG using Lipofectamine 3000 (Invitrogen, Carlsbad, CA, USA). The cells were incubated with patient sera (1:20 dilution) for 1 hour at room temperature. MOG-IgG was detected using mouse antihuman IgG1-Fc antibody conjugated with Alexa Fluor 488 (Invitrogen) in 1:500 dilution. Both assays used a semiquantitative scoring system: 0, no binding; 1, low-level binding; and 2–4, increasingly specific binding. A score of ≥1 was considered positive.

### Analysis of serum complement levels

The analysis included six complement analytes: C1q-binding circulating immune complex (CIC-C1q), C1 inhibitor (C1-INH), factor H (FH), C3, iC3b, and soluble terminal complement complex (TCC, sC5b-9). The CIC-C1q level represents the amount of complement fixing CIC that binds to immobilized human C1q protein. C1-INH and FH are the main regulators of the classical and alternative pathways, respectively. C3 plays a central role in complement system activation. Activation of either complement pathway results in the assembly of C3 convertase enzymes that cleave C3 into two fragments: C3a and C3b. iC3b is an inactivated form of C3b that is formed by the two-site cleavage of C3b by factor I with the cooperation of FH or complement receptor type 1 as cofactors. Soluble C5b-9 is nonlytic TCC and is formed when immune complexes at the C5b-7 assembly stage bind to naturally occurring regulatory serum proteins (e.g., protein S [Q8IXD4]).

The concentrations of CIC-C1q (MicroVue CIC-C1q EIA, Quidel, San Diego, CA, USA), C1-INH (MicroVue C1 Inhibitor Plus EIA, Quidel), FH (ab252359, Abcam, Cambridge, MA, USA), C3 (ab108822, Abcam), iC3b (MicroVue iC3b EIA, Quidel), and sC5b-9 (MicroVue sC5b-9 Plus EIA, Quidel) were assessed using commercially available enzyme immunoassays according to the instructions of the manufacturer.

The reference ranges for C3 and FH assays were not available, but mean levels among ten healthy adults were provided by the manufacturer: 1177 μg/ml for C3 and 288 μg/ml (range=156.1–466.5 μg/ml) for FH. For CIC-C1q, levels of at least 4.0 µg/ml are considered positive for significant levels of CIC. C1-INH concentrations less than or equal to 40% mean normal are considered significantly lower than normal (borderline, 41%–67% mean normal; normal, ≥68% mean normal). The reference values for iC3b and sC5b-9 assays were not provided.

### Statistical analysis

Group comparisons were performed using one-way ANOVA with Bonferroni post-hoc test or the Kruskal-Wallis test with post-hoc Dunn’s test for continuous variables and the chi-square test for categorical variables. Correlations among concentrations of each complement analyte and the CBA scores of either AQP4-IgG or MOG-IgG were evaluated using Pearson or Spearman’s correlation coefficients. IBM SPSS Statistics (version 22) and R software (version 4.1.0) were used for statistical analysis and data presentation. The criterion for significance was *p*<0.05.

## Results

The demographics and clinical features in the MOGAD (*N*=28), AQP4-NMOSD (*N*=53), and HC (*N*=38) groups are listed in **Table 1**. The NMOSD group was older than the HC group (*p*=0.002) and included more females than the MOGAD (65% vs 54%, *p*=0.013) and HC groups (65% vs 61%, *p*=0.019). The mean age and sex ratio did not differ between the MOGAD and HC groups. The disease duration was longer (median [IQR]=77 [11-182] months vs 3 [0.5-33] months, *p*<0.001) and disabilities were more severe (median Expanded Disability Status Scale [EDSS] score=2.5 vs 1.0, *p*=0.005) in the NMOSD group than in the MOGAD group. Three patients with NMOSD had coexisting autoimmune diseases, with two diagnosed with Sjogren syndrome and one with systemic lupus erythematosus at the time of blood sampling.

**Table 1:** Demographic, clinical features, and serum levels of complement components and regulators in study subjects.

	MOGAD			NMOSD			Controls	*p* value				
	Total (N = 28)	During attack (N = 19)	In remission (N = 9)	Total (N= 52)	During attack (N = 35)	In remission (N = 17)	(N = 38)	Total^*^	Attack^*^	Remission^*^	MOGAD	NMOSD
											A vs R	A vs R
Age, years	36.0 ± 15.3	37.8 ± 18.5	30.3 ± 9.3	44.3 ± 16.2	44.2 ± 16.6	38.2 ± 15.5	32.2 ± 4.72	0.002	0.001	0.054	0.266	0.222
Female, *N* (%)	16 (57.1)	11 (67.9)	5 (55.6)	43 (82.7)	30 (85.7)	4 (23.5)	23 (60.5)	0.021	0.030	0.443	0.907	0.409
Disease duration (month), median (range)	3.22 (0.50-32.76)	0.77 (0.23-4.57)	51.4 (29.33-101.39)	76.68 (11.33-182.34)	53.93 (6.00-171.86)	82.27 (41.01-255.49)	N/A	<0.001	<0.001	0.148	<0.001	0.181
Attack number, median (range)	3 (1-5)	2 (1-4)	5 (2.5-8)	2 (1-5)	3 (2-5)	2 (1-4)	N/A	0.943	0.111	0.066	0.016	0.170
Attack site(s)^†^							N/A					
Optic nerve	24 (85.7)	16 (84.2)	8 (88.9)	22 (42.3)	11 (31.4)	11 (64.7)		0.011	<0.001	0.357	1.000	0.476
Spinal cord	12 (42.9)	3 (15.8)	9 (100)	36 (69.2)	23 (65.7)	13 (76.5)		<0.001	<0.001	<0.001	0.530	0.770
Brain	11 (39.3)	7 (36.8)	4 (44.4)	14 (26.9)	6 (17.1)	8 (47.1)		0.924	0.181	1.000	1.000	0.494
EDSS^‡^	1.0 (0.0-2.0)	1.0 (0.0-3.0)	1.0 (0.0-2.0)	2.5 (1.25-3.75)	2.75 (1.125-4.0)	2.0 (1.25-3.0)	N/A	0.005	0.032	0.039	0.562	0.380
Use of drugs (%)	16 (57.1)	9 (47.4)	7 (77.8)	37 (71.2)	22 (62.9)	15 (88.2)	N/A					
prednisolone	11 (39.3)	8 (42.1)	3 (33.3)	25 (48.1)	17 (48.6)	8 (47.1)		0.451	0.649	0.500	0.657	0.918
Other IS	10 (35.7)	3 (15.8)^§^	7 (77.8)^§§^	30 (57.7)	17 (48.6)^§^	13 (76.5)^§§^		0.041	0.017	1.000	0.001	0.020
CBA scores of Ab	3.0 (2.0-4.0)	3.0 (2.0-4.0)	3.0 (3.0-3.5)	2.5 (1.0-3.0) ^¶^	2.5 (1.0-3.0) ^¶^	2.0 (2.0-3.0)^¶^	N/A	N/A	N/A	N/A	0.693	0.776
Complements & Regulators												
CIC-C1q (μg/ml)	84.86 ± 75.69	84.86 ± 75.69	122.01 ± 144.19	111.54 ±6.23	109.71 ± 58.25	115.32 ± 53.35	143.39 ± 122.24	<0.001	<0.001	<0.001	0.844	0.539
C1-INH (%)	105.36 ± 10.23	106.50 ± 10.57	102.95 ± 9.61	103.62 ± 21.14	98.78 ± 16.45	113.59 ± 26.32	89.65 ± 5.99	<0.001	<0.001	<0.001	0.402	0.003
C3 (μg/ml)	2030.00 ± 837.30	2196.59 ± 849.11	1678.31 ± 734.07	1972.75 691.94	2005.6 ± 746.47	1905.08 ± 578.63	1981.94 ± 116.21	0.921	0.423	0.141	0.128	0.629
iC3b (μg/ml)	34.70 ± 6.68	34.81 ± 6.31	34.45 ± 7.80	24.65 ± 8.31	26.14 ± 8.31	21.57 ± 7.63	9.30 ± 5.23	<0.001	<0.001	<0.001	0.875	0.062
FH (μg/ml)	551.27 ± 117.54	563.07 ± 129.45	526.35 ± 88.85	428.94 ±100.08	439.39 ± 91.37	407.41 ± 115.97	487.97 ± 124.53	<0.001	0.001	0.027	0.275	0.480
sC5b-9 (ng/ml)	889.59 ± 549.03	889.59 ± 549.03	N/A	1516.63 ± 933.82	1516.63 ± 933.82	N/A	838.77 ± 678.97	0.001	0.001	N/A	N/A	N/A

MOGAD, myelin oligodendrocyte glycoprotein [MOG] antibody-associated disease; NMOSD, neuromyelitis optica spectrum disorder; HC, heathy control; A, attack; R, remission; N, number; EDSS, expanded disability status scale; IS, immunosuppressants; CIC-C1q, circulating C1q-binding immune complex; C1-INH, C1 inhibitor; FH, factor H; sC5b-9, soluble terminal complement complex; Ab, antibody (anti-MOG antibody for MOGAD and anti-aquaporin4 [AQP4] antibody for NMOSD); N/A, not applicable.

^*^comparison among NMOSD, MOGAD, and HC.

^†^at the time of sampling if bloods were drawn during an attack period or during the disease course if bloods were drawn during remission.

^‡^The time interval between EDSS scoring and sampling was within 7 days for attack and 1 month for remission.

^§^For MOGAD, azathioprine (N=1, 5.3%) and mycophenolate mofetil (N=2, 10.5%) and, for NMOSD, azathioprine (N=13, 37.2%) and mycophenolate mofetil (N=4, 11.4%).

^§§^For MOGAD, azathioprine (N=3, 33.3%), mycophenolate mofetil (N=3, 33.3%), and tacrolimus (N=1, 11.1%), and, for NMOSD, azathioprine (N=11, 64.7%) and mycophenolate mofetil (N=2, 11.8%).

^¶^In NMOSD, levels of anti-AQP4 antibody at the time of assay were obtainable in 20 (during attack) and 7 (in remission) patients, respectively.

Among attack samples, more MOGAD samples were taken during optic nerve relapses (84% vs 31%, *p*<0.001) while more NMOSD samples were taken during spinal cord relapses (16% vs 66%, *p*<0.001) (**Table 1**). The proportion of patients under steroid treatment during an attack was not significantly different between the groups, but there were fewer patients who received immunosuppressive treatments (IS) in the MOGAD group than in the NMOSD group (16% vs 49%, *p*=0.017). Among attack samples on medication, 33% (*N*=3/9) of MOGAD patients and 41% (*N*=9/22) of NMOSD patients were taking oral steroids before experiencing a relapse. Additionally, blood was drawn either during or after high-dose intravenous methylprednisolone treatment in 78% (*N*=7/9) of MOGAD patients and 41% (*N*=9/22) of NMOSD patients. During the remission period, most patients were on medication, with 78% (*N*=7/9) in the MOGAD group and 88% (*N*=15/17) in the NMOSD group. At the time of relapse sampling, four NMOSD patients, previously seropositive for AQP4-IgG, showed seronegativity (CBA score of 0 or 0.5). In contrast, all MOGAD patients maintained seropositivity for MOG-IgG (CBA score of more than 1).

### Differences in complement analyte levels among MOGAD, NMOSD, and HC groups

#### During attacks

**Figure 1:**
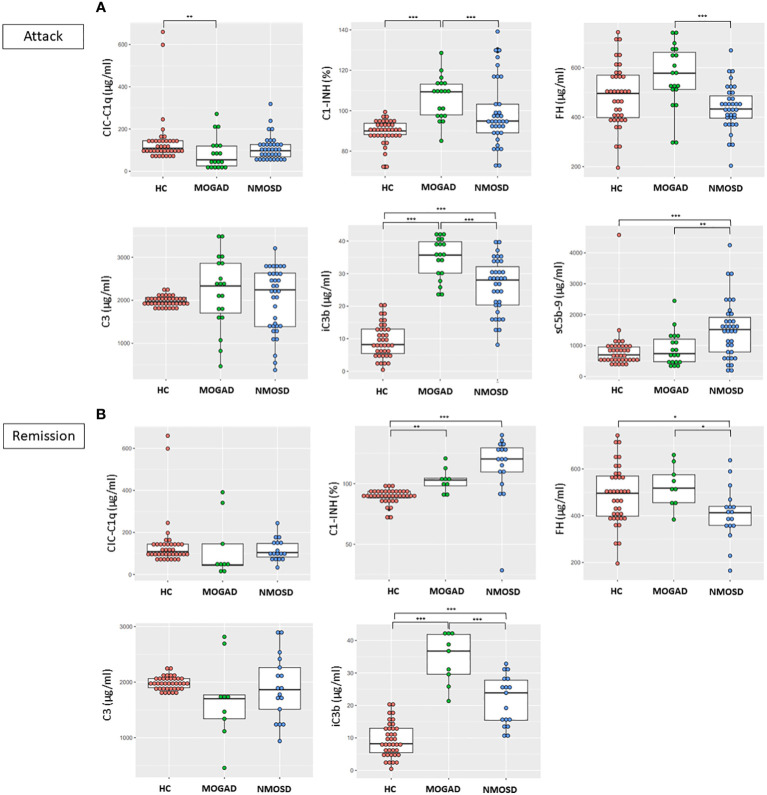
Comparisons between the concentrations of complement components and regulators in HC, MOGAD, and AQP4-NMOSD **(A)** during attacks and **(B)** remission. Each dot represents an individual sample. In the case of sC5b-9, only attack samples were assayed in MOGAD and AQP4-NMOSD. Each box plot shows the median and interquartile range. ^*^ *p*<0.05; ^**^ *p*<0.01; ^***^ *p*<0.001. CIC-C1q, circulating C1q-binding immune complex; C1-INH, C1 inhibitor; FH, factor H; sC5b-9, soluble terminal complement complex; HC, healthy control; MOGAD, myelin oligodendrocyte glycoprotein antibody-associated disease; AQP4, aquaporin4; NMOSD, neuromyelitis optica spectrum disorder.

There were higher levels of C1-INH (106.5% vs 89.6%, *p*<0.001) and iC3b (34.8 μg/ml vs 9.3 μg/ml, *p*<0.001) and lower levels of CIC-C1q (84.9 μg/ml vs 143.4 μg/ml, *p*=0.012) in the MOGAD group than in the HC group, while the levels of C3, FH, and sC5b-9 did not differ between these groups. There were higher levels of iC3b (26.1 μg/ml vs 9.3 μg/ml, *p*<0.001) and sC5b-9 (1516.6 ng/ml vs 838.8 ng/ml, *p*<0.001) in the NMOSD group than in the HC group, while the levels of CIC-C1q, C1-INH, FH and C3 did not differ between these groups (**Table 1**; **Figure 1A**). The level of sC5b-9 was significantly higher (*p*=0.006) in the NMOSD group than in the MOGAD group, while C1-INH (*p*<0.001), FH (*p*<0.001), and iC3b (*p*<0.001) were significantly higher in the MOGAD group than in the NMOSD group (**Figure 1A**).

#### During remission

There were higher levels of C1-INH (103.0% vs 89.6%, *p*=0.002) and iC3b (34.5 μg/ml vs 9.3 μg/ml, *p*<0.001) in the MOGAD group than in the HC group, while the levels of CIC-C1q, C1-INH, FH, and C3 did not differ between these groups. There were higher levels of C1-INH (113.6% vs 89.6%, *p*<0.001) and iC3b (21.6 μg/ml vs 9.3 μg/ml, *p*<0.001) and lower levels of FH (407.4 μg/ml vs 488.0 μg/ml, *p*=0.027) in the NMOSD group than in the HC group, while the levels of CIC-C1q and C3 did not differ between these groups (**Table 1**; **Figure 1B**). The levels of FH (*p*=0.013) and iC3b (*p*<0.001) were significantly higher in the MOGAD group than in the NMOSD group (**Figure 1B**).

#### Attack vs remission

Within the NMOSD group, the C1-INH level was lower during an attack than in remission (98.8% vs 113.6%, *p*=0.003). However, in MOGAD, there were no differences in the concentrations of complement components, regulators, or activation products based on disease status.

### Subgroup analyses within the MOGAD or NMOSD group, focusing on the medication status at the time of attack sampling

In the MOGAD group, 10 patients were not on medication, while 6 were on steroids alone, 1 on IS other than steroids, and 2 on a combination of both. Notably, no significant differences were observed in complement analyte levels, demographics, and clinical characteristics between MOGAD patients on and not on medication (**Supplementary Table 1**).

In the NMOSD group, 13 patients were not on medication, while 5, 5, and 12 patients were respectively on steroids alone, IS other than steroids, and a combination of both. Patients on medication showed lower levels of iC3b (*p*<0.001) and higher levels of C3 (*p*=0.008), C1-INH (*p*=0.004), and sC5b-9 (*p*<0.001) compared to those not on medication (**Supplementary Table 1**). In demographics and clinical characteristics, NMOSD patients on medication showed no significant differences when compared to those not on medication, except for a longer disease duration in the former group (97.68 [6.62-395.71] months vs 17.37 [1.22-60.52] months, *p*=0.026).

### Differences in complement analyte levels during attacks among MOGAD, NMOSD, and HC groups: subgroup analyses based on the medication status

**Figure 2:**
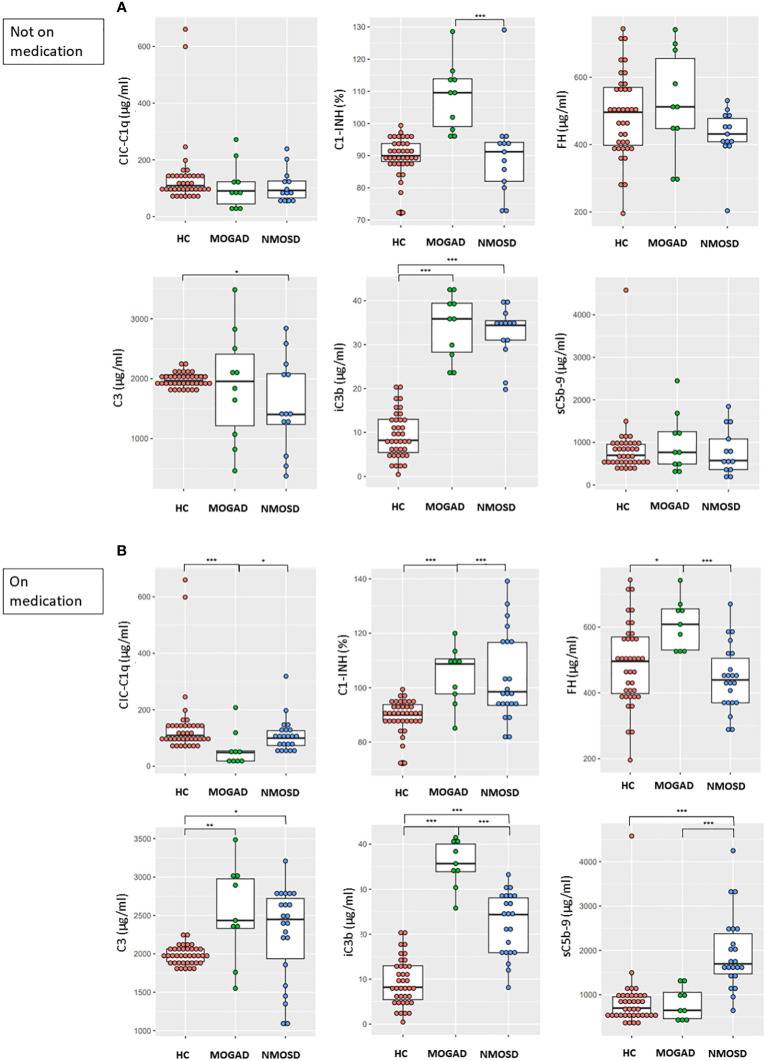
Comparisons between the concentrations of complement components and regulators in HC and **(A)** patients not on medication (MOGAD and AQP4-NMOSD) or **(B)** patients on medication (MOGAD and AQP4-NMOSD) during attacks. Each box plot shows the median and interquartile range. ^*^ *p*<0.05; ^**^ *p*<0.01; ^***^ *p*<0.001. CIC-C1q, circulating C1q-binding immune complex; C1-INH, C1 inhibitor; FH, factor H; sC5b-9, soluble terminal complement complex; HC, healthy control; MOGAD, myelin oligodendrocyte glycoprotein antibody-associated disease; AQP4, aquaporin4; NMOSD, neuromyelitis optica spectrum disorder.

MOGAD patients not on medication showed higher C1-INH and iC3b levels (both, *p*<0.001) and similar CIC-C1q, FH, C3, and sC5b-9 levels compared to HC (**Figure 2A**). NMOSD patients not on medication showed lower C3 level (*p*=0.028) and higher iC3b level (*p*<0.001) compared to HC, while CIC-C1q, C1-INH, FH and sC5b-9 levels did not differ between these groups (**Figure 2A**). Among patient groups not on medication, the MOGAD group showed a significantly higher C1-INH level (*p*<0.001) compared to the NMOSD group. There were no significant differences in CIC-C1q, FH, C3, iC3b, and sC5b-9 levels between the two disease groups.

MOGAD patients on medication showed lower CIC-C1q level (*p*<0.001) and higher C1-INH (*p*<0.001), FH (*p*=0.014), C3 (*p*=0.002), and iC3b levels (*p*<0.001) compared to HC, while sC5b-9 level did not differ between these groups (**Figure 2B**). NMOSD patients on medication showed higher C3 (*p*=0.034), iC3b (*p*<0.001), and sC5b-9 levels (*p*<0.001) compared to HC, while CIC-C1q, C1-INH, and FH levels did not differ between these groups (**Figure 2B**). Among patient groups on medication, the MOGAD group showed higher C1-INH (*p*<0.001), FH (*p*<0.001) and iC3b levels (*p*<0.001) compared to the NMOSD group, while the NMOSD group showed higher CIC-C1q (*p*=0.023) and sC5b-9 levels (*p*<0.001) compared to the MOGAD group.

### Correlations among complement analyte levels, antibody CBA scores, and EDSS scores

**Figure 3:**
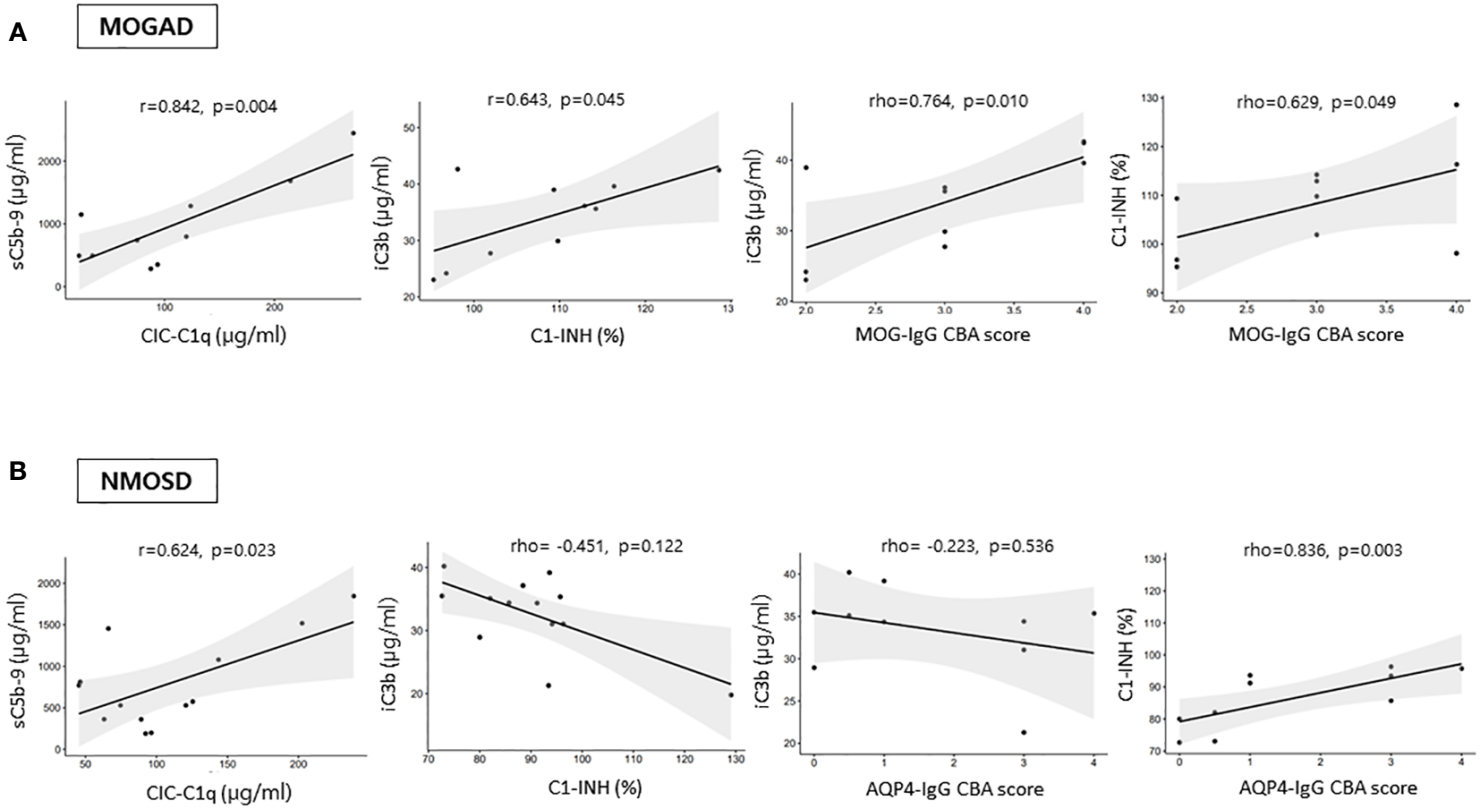
Correlations among the levels of complement analytes and antibody CBA scores during an attack in **(A)** MOGAD and **(B)** AQP4-NMOSD patients not on medication. CIC-C1q, circulating C1q-binding immune complex; C1-INH, C1 inhibitor; FH, factor H; sC5b-9, soluble terminal complement complex; MOGAD, myelin oligodendrocyte glycoprotein [MOG] antibody-associated disease; AQP4, aquaporin4; NMOSD, neuromyelitis optica spectrum disorder; CBA, cell-based assay.

In the attack samples from patients not on medication, the serum MOG-IgG CBA score had positive correlations with iC3b (*ρ*=0.764, *p*=0.010) and C1-INH (*ρ*=0.629, *p*=0.049) levels, and the AQP4-IgG CBA score had a positive correlation with C1-INH (*ρ*=0.836, *p*=0.003) (**Figure 3**). In the MOGAD group, a significant positive correlation found between C1-INH and iC3b levels (*r*=0.643, *p*=0.045), as well as between sC5b-9 and CIC-C1q levels (*r*=0.842, *p*=0.004) (**Figure 3**). The positive correlation between sC5b-9 and CIC-C1q levels (*r*=0.624, *p*=0.023) was also found in the NMOSD group (**Figure 3**). There were no significant correlations between complement analyte concentrations and EDSS scores in both the MOGAD and NMOSD groups not on medication.

In either attack or remission samples from patients on medication, no significant associations were identified among complement analytes, antibody CBA score, and EDSS score in the MOGAD group. However, positive correlations between C3 and iC3b (*ρ*=0.834, *p*<0.001 for attack samples, *ρ*=0.600, *p*=0.018 for remission samples) were identified in the NMOSD group on medication.

## Discussion

This study has demonstrated that the serum levels of complement components, regulators, and activation products differ between two antibody-mediated CNS autoimmune diseases: MOGAD and AQP4-NMOSD. During attack period, higher C1-INH, FH and iC3b levels and lower sC5b-9 level were found in the sera of MOGAD samples compared with NMOSD samples. AQP4-NMOSD patients, under medication during an attack, showed lower levels of iC3b and higher levels of C3, C1-INH, sC5b-9 compared to those not on medication. Conversely, in MOGAD, the presence of medication did not result in any significant differences in complement analytes levels. Among patients not on medication, AQP4-IgG CBA scores were positively correlated with C1-INH levels and MOG-IgG CBA scores exhibited positive correlations with C1-INH and iC3b levels. This study indicates a higher prominence of complement pathway activation and subsequent C3 degradation in MOGAD compared to AQP4-NMOSD. On the other hand, the production of TCCs was found to be more substantial in AQP4-NMOSD than in MOGAD.

Increased complement degradation product of iC3b confirmed complement pathway activation in both MOGAD and NMOSD regardless of the disease activity. Although C3 turnover increased, the C3 level did not decrease on average compared with controls. However, the concentrations of several complement analytes differed between NMOSD and MOGAD. During attacks in MOGAD, lower levels of CIC-C1q and higher levels of C1-INH were observed compared to HC. Reduced circulating C1q concentrations have been observed in several autoimmune diseases, possibly resulting from impaired synthesis or heightened consumption (12). In the context of consumption, not only are C1q levels low, but the levels of other complement components like C3 and C4 also exhibit a similar trend (12). However, this generalization may not apply to MOGAD. Increased level of C1-INH and iC3b in our patients may contribute to favoring consumption and there is a positive correlation between the level of CIC-C1q and sC5b-9. It’s noteworthy that defective production of C1q has not been reported in MOGAD. The significant reduction in CIC-C1q levels in MOGAD patients, especially in those on medication, rases the question of whether this decrease is a medication effect. While evidence suggests that ex-vivo steroid treatment enhances C1q production by macrophages (13), there is a paucity of information regarding the correlation between immunosuppressive treatments, such as azathioprine and mycophenolate mofetil, and C1q production.

Higher C1-INH, FH, and iC3b levels in MOGAD indicate more complement pathway activation compared with NMOSD. C1-INH and FH play crucial roles as primary negative regulators of complement activation, exhibiting an increase in response to inflammation (14). A recent study also found that complement system activation is a prominent feature of MOGAD (15). However, the effects of medication on C1-INH, iC3b and FH levels in both patient groups should be considered for a meaningful comparison. In NMOSD patients not on medication, iC3b levels were higher compared to those on medication, exhibiting no significant difference from MOGAD patients. Moreover, only MOGAD patients on medication showed a higher level of FH compared to NMOSD and HC. Regarding C1-INH levels, MOGAD patients consistently showed elevated C1-INH levels compared to NMOSD patients, regardless of medication status. Higher iC3b but similar FH and C1-INH levels in the blood have previously been suggested in MOG-IgG-positive NMOSD compared with AQP4-NMOSD (16). However, the interpretation of results is limited by the small sample size of MOG-IgG-positive NMOSD patients (*N*=6), the absence of information about medication, and the predominant inclusion of samples from the remission phase. Disease activities may indeed influence C1-INH levels in NMOSD, as evidenced in our study. Specifically, we observed a significantly higher C1-INH levels during remission compared to those observed during attacks.

sC5b-9 levels were elevated only in NMOSD not in MOGAD, although enhanced complement activation was suggested in both disease groups. This may suggest that complement system is strongly regulated and may play a role in the pathogenesis of MOGAD beyond TCC formation. The role of complement activation in MOGAD is less evident when compared to NMOSD. Neuropathologic studies have noted complement deposition in only a specific group of patients, prompting the exploration of alternative mechanisms underlying the pathogenicity of MOG-IgG (6, 7). A recently published experimental study also demonstrated lower ability for TCC formation in MOG-IgG compared with AQP4-IgG (17). There has been a lack of studies comparing blood complement levels between MOGAD and NMOSD, but one investigation of complement system activation found that sC5b-9 and iC3b levels were higher in MOGAD compared with HC and NMOSD (15). However, the frequently reported infectious prodrome in MOGAD may influence complement activation, as evidenced by elevated levels of sC5b-9 in the study. On the other hand, AQP4-IgG-mediated classical pathway activation and the subsequent complement-dependent cytotoxicity is a well-known pathophysiology of NMOSD (18). An anti-C5 complement inhibitor treatment has been found to be highly effective against NMOSD (19). During an attack in NMOSD, C1-INH levels were relatively lower than those during remission. In contrast, FH levels remained unchanged during an attack, comparable to HC. Our findings also suggest classical complement pathway activation associated with disease activity in NMOSD. In a study evaluating CH50 activity, indicative of total complement activity of the classical pathway, activation of complement system was observed in AQP4-NMOSD patients during attacks (20). Decreased CH50 levels during attacks or increased levels during remission were observed across different investigations (20, 21). Diminished CH50 activity during the acute phase implies potential deficiencies in complement proteins, probably due to consumption. In NMOSD patients not on medication, we noted low C3 levels, consistent with previous studies that reported low C3 and C4 levels during attacks in drug naïve AQP4-NMOSD patients (22, 23).

Acknowledging the impact of medication on complement levels, this study identified higher C3 and lower iC3b levels during attacks in medicated NMOSD patients compared to those not on medication. Steroids can inhibit the activation of the alternative amplification pathway of complement, thereby reducing the amount of C3 degradation (24). Additionally, glucocorticoids stimulate peripheral blood mononuclear cells and macrophages, leading to the upregulation of gene pathways associated with innate immunity resulting in an enhanced production of complement (25). Elevated C1-INH and sC5b-9 levels were found specifically in a patient group experiencing relapses during medication. Systemic use of glucocorticoids, known to induce a hypercoagulable state (26), plays a role in sC5b-9 generation (27). In response to this hypercoagulable state, C1-INH, a regulator for the production of coagulation factors XIIa, XIIf, and XIa, may also increase. However, it is unlikely that medication is the primary cause of variations in C1-INH and sC5b-9 levels, as similar differences were not observed in MOGAD. The longer disease duration in patients on medication (median, interquartile range [IQR]; 97.68, [6.62–396.71] months), in contrast to those not on medication (17.37 [1.22–60.52] months; p=0.026), might be a contributing factor to the elevated levels of C1-INH and sC5b-9. Recent clinical data challenge the belief that AQP4-expressing peripheral organs are typically spared from damage, revealing AQP4-IgG-associated peripheral organ damage (28). Consequently, the measured sC5b-9 levels may partially stem from peripheral organ damage in patients exposed to AQP4-IgG for a more extended duration. However, research on peripheral complement activation and its association with disease duration in NMOSD is currently limited. Undisclosed clinical factors may underlie differences in sC5b-9 levels, while features such as attack site, annual relapse rate, AQP4-IgG CBA score, and EDSS at sampling did not show any difference between patients on and not on medication in out cohort. Upon closer examination, patients not on medication showed a positive correlation between C1-INH and AQP4-IgG CBA score, comprising individuals with AQP-IgG CBA score of 0 or 0.5 (*N*=4) at sampling. Notably, this subgroup with lower AQP-IgG CBA scores also manifested the lowest levels of C1-INH and sC5b-9. A recent study, utilizing AQP-4 expressing cells, revealed a positive correlation between antibody titers and TCC levels (17). However, the replicability of this observation in peripheral blood remains uncertain and further studies are warranted.

This study had several limitations. First, the small number of samples, especially in the MOGAD group, may have reduced the statistical power and introduced unintended bias, and so caution may be needed when interpreting the results. Second, the influence of factors beyond the disease itself on the complement levels was not considered. Complement levels can be impacted by preceding infections and inflammation (29). Third, the absence of sC5b-9 levels during remission might limit the interpretation of changes in complement levels within this study. Remission phase blood samples originally intended for another study were later included in our research after analyzing attack samples. However, due to limited specimen availability, the analysis prioritized other complement concentrations over sC5b-9, leading to the absence of sC5b-9 data. Prior studies with NMOSD suggested that sC5b-9 levels were lower than those observed in HC during remission, or at least, they exhibited a decrease from the levels observed during an attack (21, 30). However, further studies utilizing paired samples from the same patients during both attack and remission phases will be necessary to corroborate our findings and validate changes consistent with disease activity. Fourth, although we employed serum samples for the analyses, the evaluation of individual complement components, particularly activation products, is better suited using plasma samples. This approach helps mitigate the risk of potential artificial complement activation (31). In addition, there was uncertainty regarding how consistent the complement analytes were during delivery because the samples were collected from multiple hospitals. For this reason, we included analytes such as iC3b and sC5b-9 that are more stable than C3a or C5a in assays. There were no differences in the concentration of each analyte according to hospital location in this study.

## Conclusion

The pattern of complement system activation in the periphery differed between AQP4-NMOSD and MOGAD. In MOGAD, more enhanced complement pathway activation with subsequent C3 degradation was indicated compared to AQP4-NMOSD. However, final product of complement pathway activation, sC5b-9, did not increase during an attack, and the levels of complement analytes did not reflect disease activity (attack versus remission). The strong regulation of the complement system suggests its potential involvement in the pathogenesis of MOGAD through mechanisms beyond the formation of the TCC. In NMOSD, we identified the expected complement system activation and the formation of TCC, more associated with classical pathway activation. Simultaneously, our study indicates that medication and autoantibody levels may influence the levels of complement analytes. Further studies with larger samples are required to augment our findings and properly identify their clinical significance.

## Data availability statement

The original contributions presented in the study are included in the article/**Supplementary Material**. Further inquiries can be directed to the corresponding authors.

## Ethics statement

The study was approved by the Institutional Review Board of Gyeongsang National University Changwon Hospital (IRB no. 2020-02-009). The studies were conducted in accordance with the local legislation and institutional requirements. The participants provided their written informed consent to participate in this study.

## Author contributions

EC: Conceptualization, Formal analysis, Funding acquisition, Methodology, Resources, Visualization, Writing – original draft. J-HM: Conceptualization, Funding acquisition, Resources, Supervision, Writing – review & editing. PW: Resources, Writing – review & editing. MJ: Investigation, Writing – review & editing. E-SJ: Investigation, Writing – review & editing. HK: Resources, Writing – review & editing. S-HK: Resources, Writing – review & editing. HS: Resources, Writing – review & editing. S-YK: Resources, Writing – review & editing. Y-ML: Resources, Writing – review & editing. S-YO: Resources, Writing – review & editing. HL: Resources, Writing – review & editing. ES: Resources, Writing – review & editing. S-SL: Resources, Writing – review & editing. JO: Resources, Writing – review & editing. SK: Resources, Writing – review & editing. S-YH: Resources, Writing – review & editing. J-YC: Resources, Writing – review & editing. JS: Resources, Writing – review & editing. B-JK: Resources, Writing – review & editing. BK: Conceptualization, Funding acquisition, Resources, Supervision, Writing – review & editing.
