# Supplementary material for: Differentiated pattern of complement system activation between MOG-IgG-associated disease and AQP4-IgG-positive neuromyelitis optica spectrum disorder
Source: Front Immunol. 2024 Mar 21;15:1320094. doi: 10.3389/fimmu.2024.1320094 (PMC10991751; doi:10.3389/fimmu.2024.1320094)
Supplement: Supplementary file 1 [file DataSheet_1.pdf]

Supplementary Table 1 Comparisons of demographic, clinical features, and serum levels of complement components and regulators during the acute phase: impact of medication status at sampling in MOGAD and NMOSD

	MOGAD			NMOSD		
At the time of sampling during the acute phase	Not on medication (*N*=10)	On medication (*N*=9)	*p* value	Not on medication (*N*=13)	On medication (*N*=22)	*p* value
Age	40.72 ± 17.37	34.5 ± 20.1	0.482	43.5 ± 17.3	44.6 ± 16.5	0.849
Female, *N* (%)	6 (60.0)	5 (55.6)	1.000	10 (76.9)	20 (90.9)	0.337
Disease duration (month), median (range)	0.42 (0.15−8.71)	1.13 (0.67−3.22)	0.447	17.37 (1.22−60.52)	97.68 (6.62−395.71)	0.026
Attack number, median (IQR)	2 (1−5)	2 (1−4)	0.968	3 (1−5)	3 (2−7)	0.489
Elapsed time since symptom onset (days), median (IQR)	6 (3.75−11.75)	12 (6.5−24)	0.054	5.5 (2−15)	8 (3−15)	0.589
Attack site(s)†						
Optic nerve	10 (100)	6 (66.7)	0.087	3 (23.1)	8 (36.4)	0.478
Spinal cord	2 (20.0)	1 (11.1)	1.000	11 (84.6)	12 (54.5)	0.139
Brain	4 (40.0)	3 (33.3)	1.000	2 (15.4)	4 (18.2)	1.000
EDSS‡	1.0 (0.0−3.0)	2.0 (0.0−3.0)	0.905	1.25 (0.75−4.5)	3.0 (2.0−5.0)	0.192
Complements & Regulators						
CIC-C1q (μg/ml)	106.65 ± 81.60	60.64 ± 64.40	0.113	108.03 ± 58.53	100.70 ± 59.44	0.898
C1-INH (%)	108.34 ± 10.45	104.45 ± 10.94	0.439	90.42 ± 14.18	103.72 ± 15.95	0.008
C3 (μg/ml)	1885.84 ± 930.70	2541.87 ± 626.82	0.093	1556.12 ± 767.45	2271.24 ± 605.48	0.004
iC3b (μg/ml)	34.02 ± 7.32	35.70 ± 5.26	0.578	32.56 ± 6.20	22.35 ± 7.03	<0.001
iC3b/C3	0.024 ± 0.016	0.015 ± 0.004	0.156	0.028 ± 0.020	0.0099 ± 0.0019	<0.001
FH (μg/ml)	521.84 ± 156.34	608.89 ± 75.64	0.148	428.41 ± 80.57	445.87 ± 98.44	0.592
sC5b-9 (ng/ml)	972.36 ± 684.12	797.64 ± 365.56	0.504	786.29 ± 534.50	1948.21 ± 850.45	<0.001
CBA scores of Ab	3.0 (2.0−4.0)	3.0 (2.0−4.0)	0.842	1.0 (0.5−3.0)§	3.0 (2.0−3.0)§	0.165

MOGAD, myelin oligodendrocyte glycoprotein [MOG] antibody-associated disease; NMOSD, neuromyelitis optica spectrum disorder; HC, heathy control; A, attack; R, remission; N, number; EDSS, expanded disability status scale; IS, immunosuppressants; CIC-C1q, circulating C1q-binding immune complex; C1-INH, C1 inhibitor; FH, factor H; sC5b-9, soluble terminal complement complex; Ab, antibody (anti-MOG antibody for MOGAD and anti-aquaporin4 [AQP4] antibody for NMOSD); IQR, interquartile range; N/A, not applicable

†at the time of sampling

[ǂ]The time interval between EDSS scoring and sampling was within 7 days

[§]In NMOSD, CBA scores for anti-AQP4 antibody were available for both 10 patients not on medication and 10 patients on medication at the time of the assay
